# Identification of Competing Endogenous RNAs (ceRNAs) Network Associated with Drought Tolerance in *Medicago truncatula* with Rhizobium Symbiosis

**DOI:** 10.3390/ijms232214237

**Published:** 2022-11-17

**Authors:** Jiaxian Jing, Peizhi Yang, Yue Wang, Qihao Qu, Jie An, Bingzhe Fu, Xiaoning Hu, Yi Zhou, Tianming Hu, Yuman Cao

**Affiliations:** 1 College of Grassland Agriculture, Northwest A&F University, Xianyang 712100, China; 2 State Key Laboratory of Agrobiotechnology, College of Agronomy and Biotechnology, China Agricultural University, Beijing 100083, China; 3 School of Agriculture, Ningxia University, Yinchuan 750021, China; 4 Shaanxi Academy of Forestry, Xi’an 710082, China; 5 School of Agriculture Food and Wine, The University of Adelaide, Waite Campus, Urrbrae, SA 5064, Australia

**Keywords:** ceRNA network, drought stress, *Medicago truncatula*, miR169l, nodules

## Abstract

Drought, bringing the risks of agricultural production losses, is becoming a globally environmental stress. Previous results suggested that legumes with nodules exhibited superior drought tolerance compared with the non-nodule group. To investigate the molecular mechanism of rhizobium symbiosis impacting drought tolerance, transcriptome and sRNAome sequencing were performed to identify the potential mRNA–miRNA–ncRNA dynamic network. Our results revealed that seedlings with active nodules exhibited enhanced drought tolerance by reserving energy, synthesizing N-glycans, and medicating systemic acquired resistance due to the early effects of symbiotic nitrogen fixation (SNF) triggered in contrast to the drought susceptible with inactive nodules. The improved drought tolerance might be involved in the decreased expression levels of miRNA such as mtr_miR169l-5p, mtr_miR398b, and mtr_miR398c and its target genes in seedlings with active nodules. Based on the negative expression pattern between miRNA and its target genes, we constructed an mRNA–miR169l–ncRNA ceRNA network. During severe drought stress, the lncRNA alternative splicings *TCONS_00049507* and *TCONS_00049510* competitively interacted with mtr_miR169l-5p, which upregulated the expression of NUCLEAR FACTOR-Y (NF-Y) transcription factor subfamily NF-YA genes *MtNF-YA2* and *MtNF-YA3* to regulate their downstream drought-response genes. Our results emphasized the importance of SNF plants affecting drought tolerance. In conclusion, our work provides insight into ceRNA involvement in rhizobium symbiosis contributing to drought tolerance and provides molecular evidence for future study.

## 1. Introduction

Drought has become a global issue resulting in a decrease of crop production and enormous economic loss (e.g., USD 9.6 billion in the USA per year) [1]. Drought stress has significant effects on the phenome, transcriptome, proteome, and metabolome of plants [2]. When sensing drought, plant cells reconfigure a new homeostasis through numerous biological processes [3]. For example, various miRNAs and their target genes were participated in the regulation of homeostasis reconstruction [4]. It was reported that overexpression of miR156 improved drought tolerance by partially silencing target gene *SPL13* through accumulation of osmoprotective compounds proline, ABA, and antioxidants in alfalfa [5]. In tomato, decrease of sly-miR159 promoted drought tolerance by the increase of SlMYB33 transcript correlated with accumulation of the proline and putrescine [6].

Symbiotic nitrogen fixation (SNF) is a nitrogen-fixation system based on legume-rhizobia symbiosis, and can convert atmospheric N_2_ into nitrate nitrogen (NO_3_^−^-N) and ammonium nitrogen (NH_4_^+^-N) [7].The SNF includes a series processes, e.g., triggering nodulation signal transduction, symbiosis selection formation, and plant defense inhibition [8]. Our early study showed that nodulation and the formation of active nodules could enhance drought tolerance in alfalfa by reducing lipid peroxidation, and increasing free proline and expansible sugar under drought stress [9]. Moreover, rhizobium symbiosis improved the tolerance of alfalfa under short-term salt stress [10]. The drought tolerant soybean cultivar DT2008 was characterized by better nodule development than the drought susceptible cultivar W82 [11]. Previous studies focused on the physiological mechanisms involved in rhizobium symbiosis contributing to stress tolerance of plants, but little is known about the molecular mechanism of rhizobium symbiosis impacting drought tolerance.

Non-coding RNAs were involved in many biological processes in plants, such as reproductive development [12], positive regulation of the expression of adjacent genes [13], as well as response to biotic stress [14], and abiotic stress [15]. The ncRNAs could be divided into three types: (1) long noncoding RNAs (lncRNAs), with lengths over 200 nt (unit of single-strand base number); (2) circular RNAs (circRNAs), with closed circular structure and not affected by RNA exonuclease; (3) small noncoding RNAs (sRNAs), which were composed of microRNAs (miRNAs) and small-interfering RNAs (siRNAs) [16]. In addition, some research indicated that circRNAs acted as miRNA decoys [17], protein scaffolds [18], and protein sequestrators [19] in mammals. The miRNA genes were transcribed into primary-miRNA (pri-miRNA) by RNA polymerase II (Pol II), subsequently processing pri-miRNA to precursor-miRNA (pre-miRNA) which contains stem-loop structures by Dicer-like protein 1 (DCL1) [20]. Then, the DCL protein cleaved pre-miRNA into an miRNA duplex, which finally was produced into miRNA [16]. The RNA-induced silencing complex (RISC), formed by Argonaute (AGO) protein with miRNA, enabled to recognize miRNA response elements (MREs) and then repress miRNA target genes via cleavage or translation inhibition [20,21]. The mRNA, lncRNA, and circRNA, which contained MRE(s), were called miRNA sponges, as they could be repressed by RISC [17].

The mRNA, lncRNA, and circRNA were named as competing endogenous RNAs (ceRNAs) as they competitively combine with the same miRNA [21]. For instance, the *SPL2-like/SPL33*–miR156a–*MLNC3.2/MLNC4.6* network was involved in the regulations of apple (*Malus domestica*) fruit pigment [22]. With more lncRNA *MLNC3.2/MLNC4.6* as miR156a sponge under white or blue light, anthocyanin was accumulated in apple fruit by improving the expression of mRNA *SPL2-like/SPL33* [22]. Furthermore, *NBS-LRR*–miR482a–*lncRNA15492*, a ceRNA network, responded to biotic stress in plants [23]. The mRNA *NBS-LRR,* which positively regulated tomato (*Solanum lycopersicum*) resistance to *Phytophthora infestans,* was cleaved by miR482a, whereas *lncRNA15492* inhibited precursor miR482a expression through antisense strands of lncRNAs [23]. In rice, overexpressing lncRNA *TCONS_00021861* could be competitively combined with miR528-3p, which released *YUCCA7*, the miRNA target gene, to active IAA biosynthetic pathway and confer resistance to drought stress [24]. However, there was little research on the ceRNA network in legumes with rhizobium symbiosis contributing to drought stress.

*Medicago truncatula*, with the characteristics of a short lifecycle, a small genome size (419 Mb, 2n = 16), and self-pollination, is a model leguminous plant for the study of nodule nitrogen fixation, especially the drought tolerance associated with rhizobium symbiosis. Previous study showed that legumes with nodules exhibited superior drought tolerance compared with the control (without nodules) treatment. However, the ceRNA regulatory network of nodules contributing to the drought tolerance of *M. truncatula* is still unclear. To investigate the regulatory mechanism of ceRNA, transcriptome and sRNAome sequencing were performed to identify the potential mRNA–miRNA–ncRNA dynamic network. Our result showed that as the water deficiency continues, *M. truncatula* in active nodule (AN) treatment medicated systemic acquired resistance due to early effects triggered by SNF. The mRNA–miR169l–ncRNA network contributed to the drought tolerance by its target genes competitively combined with mtr_miR169l-5p.

## 2. Result

### 2.1. Morphology of M. truncatula with Nodules under Drought Treatment

**Figure 1:**
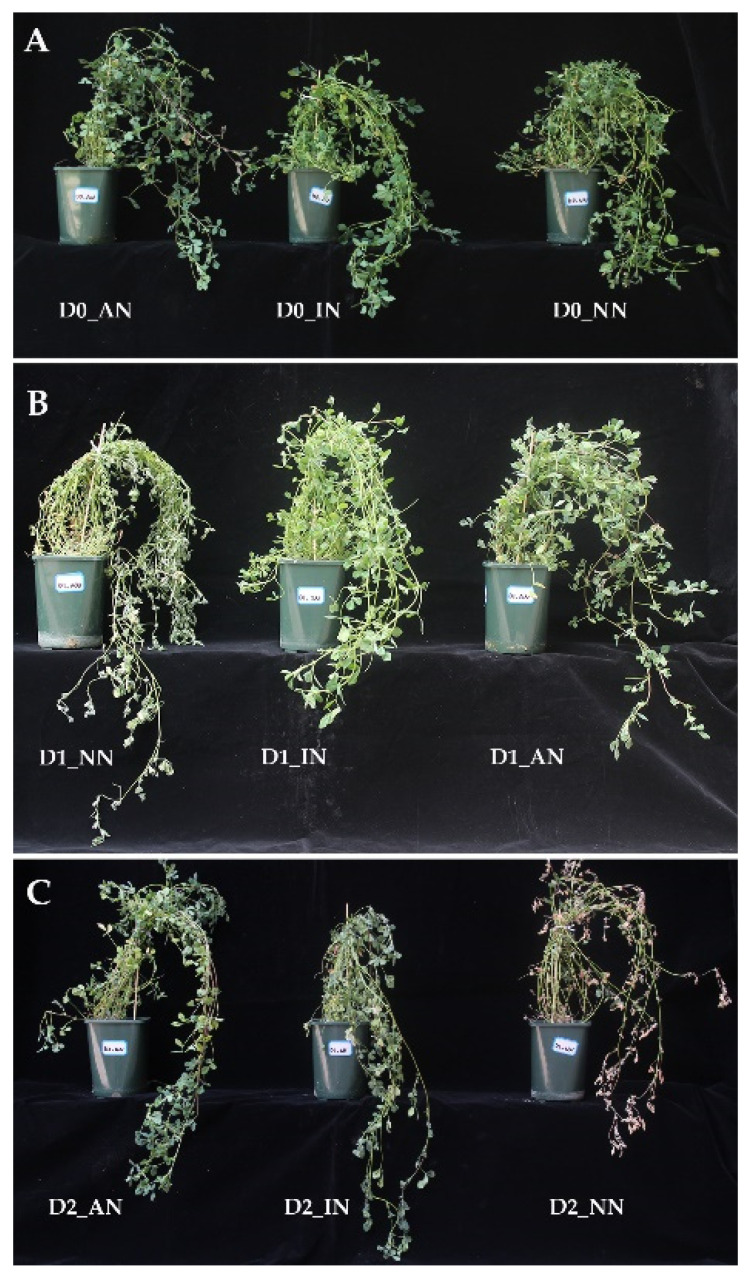
Phenotypic comparison of plants with no nodules (NN), inactive nodules (IN), active nodules (AN) under different drought treatments. Phenotypes of NN, IN, AN plants under (**A**) normal condition (D0); (**B**) mild drought stress (D1); (**C**) severe drought stress (D2).

*M. truncatula* inoculated with rhizobium formed nodules in the root. During our early research, the active nodules were pink with nitrogen fixation ability, while the inactive nodules were white without (or barely with) nitrogen fixation ability [10]. With different drought and nodule treatments, 27 samples divided with nine grouping treatments, with three biological duplications (Table 1). Without drought stress (D0), the aboveground plants exhibited no difference in active nodule (AN), inactive nodule (IN), and no nodule (NN) treatment (Figure 1A). However, under mild drought stress (D1), leaves of D1_NN were easier to wilt than IN and AN plants (Figure 1B). Under severe drought stress (D2), the AN plants were more tolerant to drought stress compared with IN and NN plants (Figure 1C). It showed that nodulation (AN and IN) could improve the resistance when suffering from soil water deficit stress.

### 2.2. Transcriptome Sequencing Feature Analysis

Under different treatment, 27 samples were sequencing of transcriptome and sRNAome to investigate the molecular regulation mechanism of nodules impacting drought tolerance (Table S1). On average, 7.48 Gb of data was obtained for each sample after quality control and filtration with an average base of Q20 > 96.4% and the Q30 > 90.9%.

**Figure 2:**
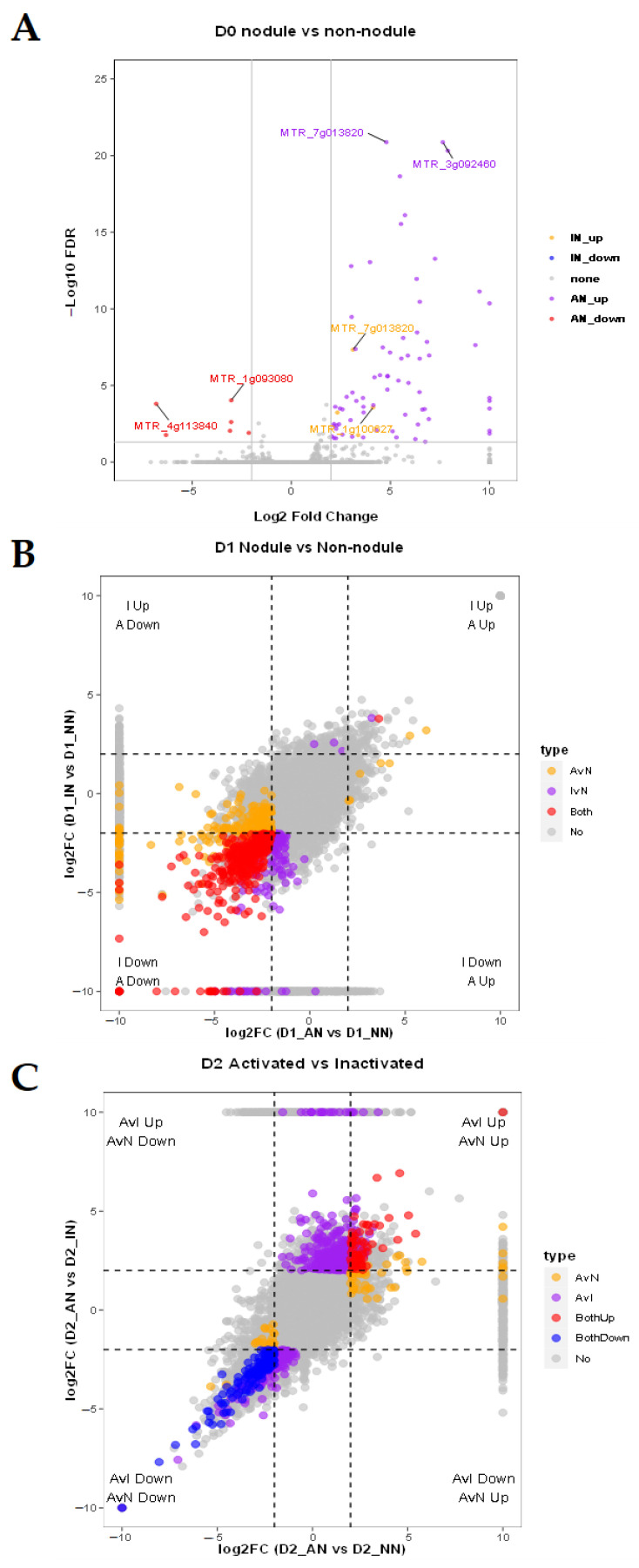

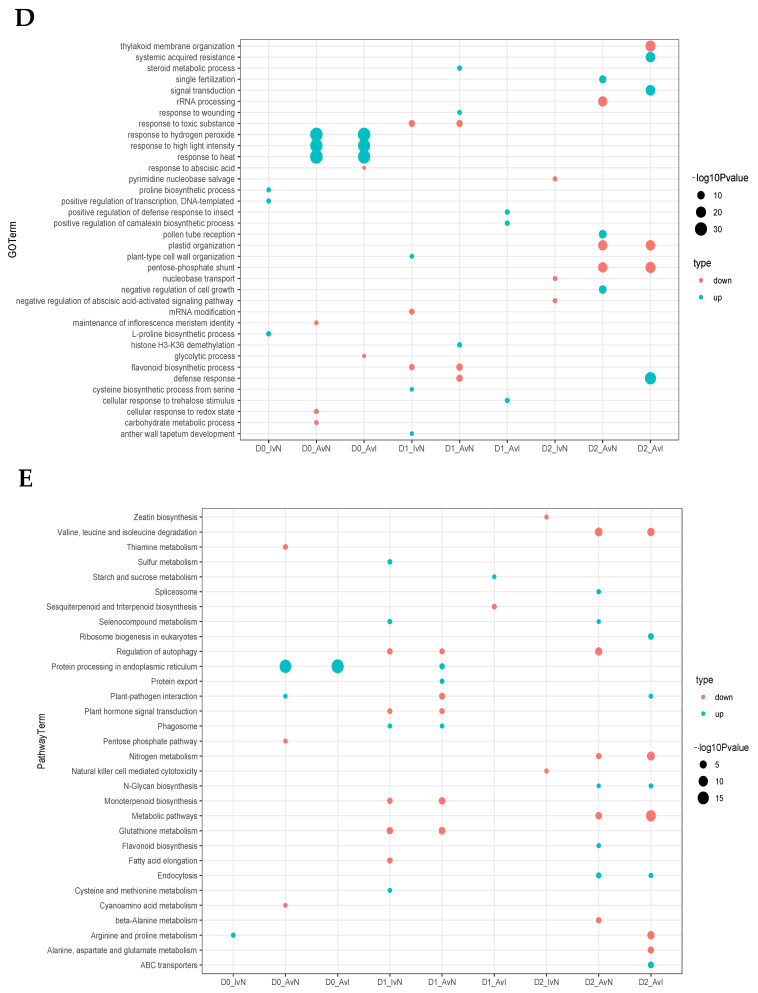
Analysis of mRNA expression feature of plants with no nodules (NN), inactive nodules (IN), active nodules (AN) under normal condition (D0), mild drought stress (D1), severe drought stress (D2). (**A**) DEGs expression level of D0_AN vs. D0_NN, D0_IN vs. D0_NN under D0. Gene locus tags were annotated for the top two with the lowest FDR values for each set except “none” group; (**B**) under the condition of D1, DEGs expression level of D1_AN vs. D1_NN, D1_IN vs. D1_NN; (**C**) under the condition of D2, DEGs expression level of D2_AN vs. D2_NN, D2_AN vs. D2_IN; (**D**) GO analysis of biological process and (**E**) KEGG analysis under nodulation and drought treatments. All the colored DEGs were filtrated with FDR < 0.05. It was artificially stipulated that the threshold value is 10/−10 when the log2 fold change value tends to be infinite. All the GO terms and pathways were filtrated with *p*-value < 0.05. Top 3 GO terms/pathways in upregulated or downregulated regulation module for each sample with the lowest *p*-value were selected.

Without drought stress, the D0_IN vs. D0_NN only had four differentially expressed genes (DEGs) (Table S2). While compared with D0_AN, the number of DEGs in uninoculated (D0_NN) and inactivated (D0_IN) conditions were 86 and 73, respectively (Figure 2A). Intriguingly, the *MTR_7g013820* gene putatively encoding NINJA family protein AFP3 was the only upregulated DEG detected in D0_IN vs. D0_NN, D0_AN vs. D0_NN, and D0_AN vs. D0_IN. It showed that AFP3 might be upregulated in IN and have the highest expression level in AN plants. Under the D1 condition, the DEGs of D1_IN vs. D1_NN, D1_AN vs. D1_NN, D1_AN vs. D1_IN were 768 (16 upregulated and 752 downregulated), 988 (24 upregulated and 964 downregulated), and 12 (7 upregulated and 5 downregulated), respectively. Most of the DEGs shared between D1_IN vs. D1_NN, and D1_AN vs. D1_NN were downregulated in the mild drought stress (Figure 2B). It indicated that the nodulated plants would respond to drought stress by downregulating genes.

In the severe drought stress (D2) condition, there were 38 DEGs (downregulated) of D2_IN vs. D2_NN, 710 DEGs (290 upregulated and 420 downregulated) of D2_AN vs. D2_NN, and 2158 DEGs (1087 upregulated and 1071 downregulated) of D2_AN vs. D2_IN. DEGs shared between D2_AN vs. D2_NN, and D2_AN vs. D2_IN showed that AN plants had specific genes to respond long term drought tolerance (Figure 2C). It seemed that AN plants were enabled to regulate more unique genes than IN plants (purple points in Figure 2C), and these DEGs contributed to improving persistent resistance to drought stress in AN plants compared with IN plants.

To describe the function of DEGs, gene ontology (GO) enrichment analysis was used to classify and annotate DEGs Figure 2D and Figure S1, Table S3). With D0 treatment, the most enriched GOs from IN vs. NN were ‘L-protine/protine biosynthetic process’ (GO: 0055129, 0006561) and ‘positive regulation of transcription’ (GO: 0045893). The most enriching GO terms were ‘response to heat’ (GO: 0009408), ‘response to hydrogen peroxide’ (GO: 0042542), and ‘response to high light intensity’ (GO: 0009644) in AN plants compared with NN or IN. Under the D1 condition, the most enriched GO terms in common with IN and AN plants compared with NN plants were ‘response to toxic substance’ (GO: 0009636), ‘mRNA modification’ (GO: 0016556), and ‘flavonoid biosynthetic process’ (GO: 0009813), while unique enriched GO term in AN plants compared with NN was ‘defense response’ (GO: 0006952). Differently from previous GO terms, the most enrichment GO terms in AN plants against IN plants were ‘histone H3-K36 demethylation’ (GO: 0070544), ‘positive regulation of camalexin biosynthetic process’ (GO: 1901183), and ‘defense response to insect’ (GO: 1900367). With D2 treatment, the unique enriched GO terms in AN plants were ‘plastid organization’ (GO: 0009657), ‘rRNA processing’ (GO: 0006364), and ‘pentose-phosphate shunt’ (GO: 0006098). The IN and AN plants shared some biological processes with the similar expression tendency in D1 condition, while most processes in AN plants specifically expressed under D2 condition. The similar GO terms and phenotype performances of IN and AN in D1 treatments are discussed in the Discussion.

To comprehend how nodules affected the pathway regulation of plants, we introduced KEGG (Kyoto Encyclopedia of Genes and Genomes) to investigate pathways triggered by nodulation during the regulation of abiotic stress (Figure 2E, Table S4). In the D0 condition, the IN plants participated in arginine and proline metabolism, while the AN plants were mainly involved in protein processing in the endoplasmic reticulum, which was a unique upregulated pathway in AN. With D1 treatment, nodulation treatment (IN and AN) was involved in down-regulating glutathione metabolism, regulation of autophagy, monoterpenoid biosynthesis, and up-regulating phagosome pathways. Sesquiterpenoid and triterpenoid biosynthesis pathways were downregulated in AN plants in contrast to IN. Under D2, the IN plants participated in natural killer cell-mediated cytotoxicity and zeatin biosynthesis pathway. The AN plants were especially involved in the downregulating process of valine, leucine, and isoleucine degradation, regulation of autophagy, metabolic pathways, and nitrogen metabolism. Moreover, the unique upregulation processes, such as endocytosis and N-glycan biosynthesis, were enriched in AN plants in the D2 condition. The gene expression level of cell autophagy in AN was lower than IN and NN in the D2 condition. Our result showed that AN reduced energy loss to improve drought tolerance through downregulating metabolism and nitrogen metabolism processes.

### 2.3. Different Expression Patterns Analysis of mRNA

**Figure 3:**
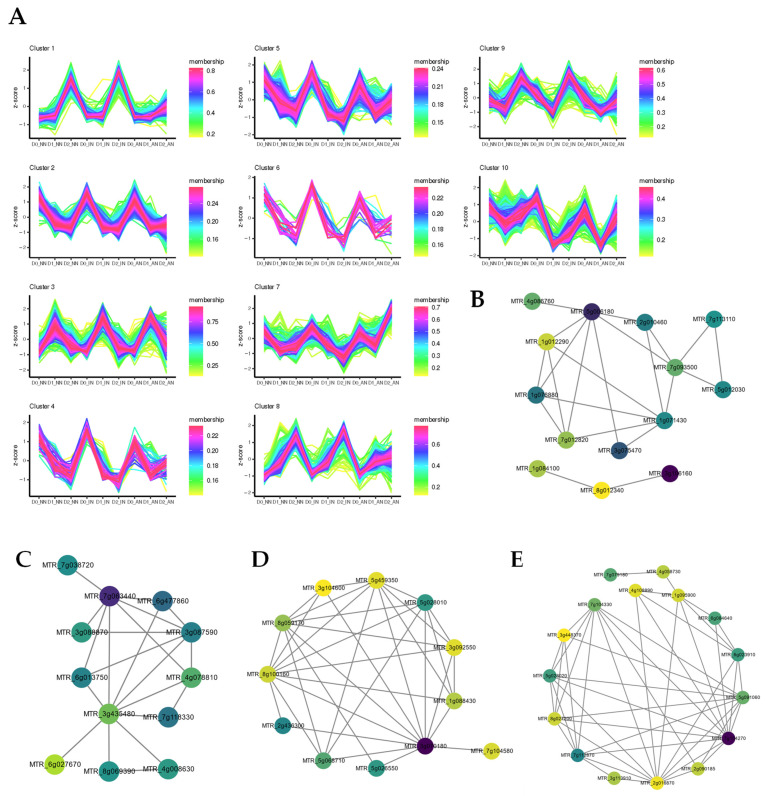
Expression patterns analysis of DEGs. (**A**) cluster prediction analysis; (**B**–**E**) hub genes networks in WGCNA analysis of (**B**) green module relative to D1 (**C**) pink module relative to D1; (**D**) blue module relative to D2; (**E**) hub gene network of yellow module relative to D2. The lines in were the mapping of the filter weight ≥ 0.2 except pink module with filter weight belonging to [0.15, 0.197]. The color of hub genes was mapped to the MCC algorithm-calculated results. To identify more underlying relationships between traits and DEGs, WGCNA was performed to identify crucial genes relative to drought and nodulation. A total of 24724 DEGs were used for subsequent analysis after iterative filtering of genes with too many missing entries. Evaluation parameters of scale-free networks were calculated to figure out the soft threshold at 24, according to the constructed gene co-expression network (Figure S2). Most of the DEGs were classified into 24 modules except for the grey module with disabled categorized genes. The network heatmap was plotted to exhibit an expression cluster with all DEGs and a hierarchical cluster with different modules (Figure S3). Modules with biological significance associated with traits were singled out through correlation coefficients between modules and various phenotypes (Figure S4). Hub genes in each module that were considered as potential critical regulation nodes were selected (**B**–**E**). DEGs in green and pink modules were related to D1 treatment, and in blue and yellow modules were correlated with D2 treatment. *MTR_5g006180* and *MTR_7g063440* were the hub genes involved with D1 treatment. *MTR_3g071080* and *MTR_7g104270* were predicted to be the hub regulated genes in D2 treatment.

The mRNA cluster prediction and weighted gene co-expression network analysis (WGCNA) were performed to identify the expression pattern between nodulation and drought. The R package TCseq (https://bioconductor.org/packages/TCseq/, accessed on 20 August 2021) was used to identify the DEGs expression patterns. All the DEGs were divided into 10 expression clusters (Figure 3), which could be segmented into four types based on the expression patterns. (type 1) In clusters 2, 3, and 10, the DEGs expression of IN and AN plants during drought stress showed a similar pattern. The DEGs that were located at chloroplast associated with protein process, abiotic stimulate response, inositol phosphate metabolism, and circadian rhythm were classified into type 1. (type 2) DEGs in clusters 1,8 and 9 were upregulated in the AN group. In D2, most DEGs fitting the patterns exhibited slight upregulation in AN treatment compared with NN and IN. DEGs enriched in type 2 were mainly focused on the response to water deprivation, cell division, hormone, autophagy, and nitrogen metabolism. (type 3) In clusters 4,5 and 6, DEGs exhibited first downregulation and then upregulation in sustained drought stress in AN, while DEGs of NN and IN plants were downregulated during drought stress. (type 4) In cluster 7, different treatments of nodulation displayed various expression tendencies. The DEGs in cluster 7 were downregulated when initial drought and subsequently plants showed diverse expression patterns, with NN group mildly upregulated, IN group downregulated, and AN group sharply upregulated (Figure 3A, Table S5). DEGs in type 4 were mainly involved in nuclear ribosome biogenesis and RNA degradation.

### 2.4. Feature Analysis and Expression Patterns of ncRNAs

**Figure 4:**
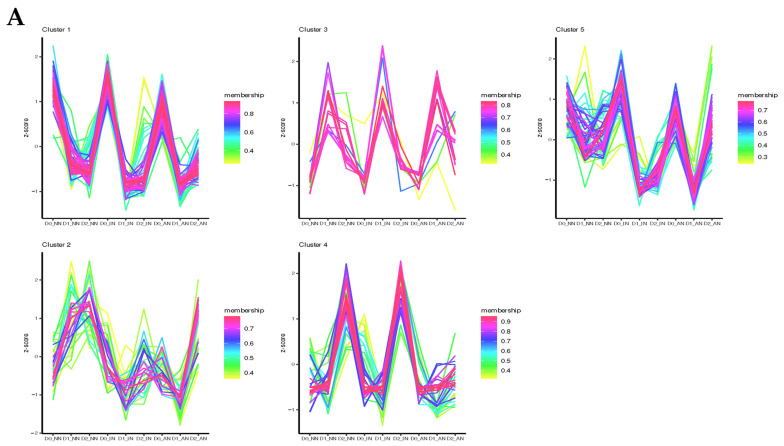

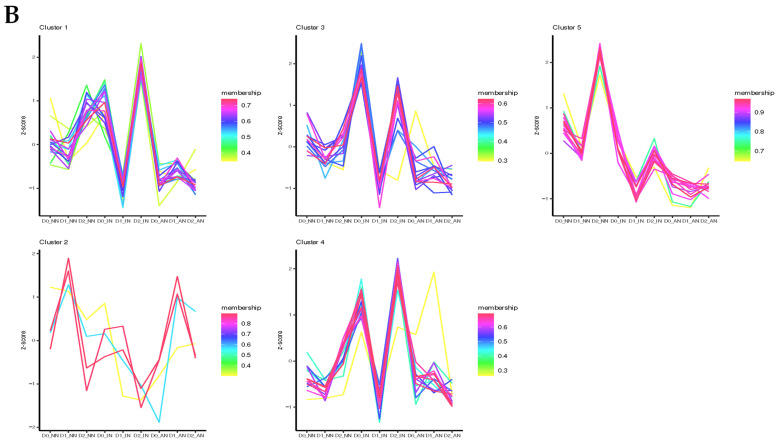
Cluster prediction analysis of (**A**) differentially expressed lncRNAs (DElncRNAs) and (**B**) differentially expressed microRNAs (DEmiRNA). With a total of 740 circRNAs obtained from 27 samples of 9 treatments, 122 differentially expressed circRNAs (DEcircRNAs) were identified to investigate the candidates of potential ceRNAs (Table S8). In D0 conditions, of the 68 DEcircRNAs, 8 DEcircRNAs showed different expression levels among diverse nodulation treatments, and 10 DEcircRNAs were predicted as the targets of miRNAs. The 74 DEcircRNAs were obtained within the D1 treatment, among which 7 DEcircRNAs were potential ceRNAs candidates. Under the D2 treatment, 94 DEcircRNAs were detected with 5 DEcircRNAs predicted as negatively regulated targets of differentially expressed miRNAs (DEmiRNAs). DEcircRNA cluster analysis was performed to describe DEcircRNAs expression patterns of rhizobium symbiosis contributing to drought tolerance (Figure S5, Table S9).

Computerized predicted lncRNAs were mapped to the *M. truncatula* genome, with an average mapping rate of 3%. Without drought stress treatment (D0), all the differentially expressed lncRNAs (DElncRNAs) detected in AN plants were upregulated compared with NN and IN plants. On the contrary, in D1 treatment DElncRNAs of IN and AN plants were downregulated except *TCONS_00002192*. *TCONS_00002192* was the unique gene upregulating in AN plants contrasted with IN or NN plants. Under D2 treatment, only a few downregulated DElncRNAs were identified in AN and IN plants compared with NN plants. Considering AN vs. IN, 57 DElncRNAs (15 upregulated and 42 downregulated) were identified (Table S6). DElncRNA-cluster analysis was performed to investigate hub regulative lncRNAs for the nodulation treatment impacted by drought stress (Figure 4A, Table S7). Intriguingly, hub DElncRNAs in clusters 4 and 5 could be potential candidates involved in activated nodule regulation with drought stress.

The 3246 miRNAs were identified from all the 27 samples, with the average mapping rate of 98.1%. All the DEmiRNAs were shown in a heatmap with diverse treatments (Figure S6). In D0, mtr-miR2111m-3p was differentially expressed both in IN and AN plants, which demonstrated that mtr_miR2111m-3p was upregulated in rhizobium infection, no matter that the nodules were active or inactive (Table S10). The miR2111 acted as a positive regulator of rhizobium infection and was subsequently repressed by the HAR1 receptor after infection in leaves [25,26]. The Mtr-miR408-3p, mtr-miR398b, and mtr-miR398c specifically downregulated genes expression at AN plants compared with IN and NN plants. Within D1 treatment, mtr-miR2111f is distinctively downregulated in AN plants. Meanwhile, mtr-miR2618b was upregulated in nodulation treatment (both IN and AN). Under D2 treatment, mtr-miR2111o, mtr-miR2111j, mtr-miR2111c, and mtr-miR2111f were downregulated in seedlings with nodules (IN and AN). However, mtr-miR5260 and mtr-miR5215 were only downregulated in AN treatment. Series cluster analysis was applied to observe the expression tendency of DEmiRNAs (Figure 4B, Table S11). Remarkably, DEmiRNAs exhibited lower expression among all the drought stress in AN treatment. Therefore, our results indicated that several miRNAs involved in the SNF process (AN plants) might participate in drought stress tolerance.

### 2.5. Analysis of miRNA-Target Genes

One of the ceRNA regulation networks was coding RNAs and non-coding RNAs competitions of shared miRNAs. Identifying the miRNA-target genes was of crucial importance in investigating the regulatory network of ceRNAs. The psRNAtarget [27] was preformed to identify the potential MRE sites between miRNAs and their target genes. The target genes, which consist of mRNAs and lncRNAs, competingly interacted with miRNAs. Within D0 treatment, there was no mRNA negatively regulated by miRNA identified from all the DEGs of D0 treatment. Compared to the whole *M. truncatula* reference genome, the target genes of DEmiRNAs were identified as MYB family transcription factors. Within D1 treatment, 5 DEmiRNAs were identified interacting with different target DEGs, among which miR2608 was negative regulation with its target gene (*Medtr0011s0020*). Under D2 treatment, eight DEmiRNAs, of which miR159b, miR169l-5p, miR397-5p, miR5747, and miR5291b were negatively regulated with their target DEGs, were predicted to interact with target DEGs (Table 2). Moreover, we selected the hub genes of cluster analysis to look up for candidate MRE loc site. Mtr-miR1510a-5p, regarded as a target NB-LRR domain gene and concerning resistance to *Phytophthora sojae* in soybean [28], was recognized (Table S12).

With D0 treatment, 13 DElncRNAs, some of which had more than one MREs, were identified as the potential miRNA target genes (Table S12). MiR397-5p, miR5248, miR408-3p, miR5215, and miR2661 were negatively regulated with the target DElncRNAs (Table 2). Under D1 treatment, 17 DEmiRNAs were found in 76 target DElncRNAs and only miR2608 was negatively regulated with its 8 target DElncRNAs. With D2 treatment, 48 DElncRNAs, with a total of 30 different MREs were identified as the miRNA target genes. Moreover, 13 DElncRNAs from the 48 DElncRNAs were distinguished to be negatively regulated by 11 DEmiRNAs. Through searching for MREs in hub lncRNA with cluster analysis, mtr-miR2111m-3p, mtr-miR160c, mtr-miR397-5p, mtr-miR5248, mtr-miR5215, as well as mtr-miR395a were identified regulating hub lncRNAs in the cluster.

From the sequencing data, there was no DEcircRNA regarded as DEmiRNA target genes in D0 and D1 treatment. With D2 treatment, 19 circRNAs were identified with various MREs (Table 2). Intriguingly, all the miRNAs, miR5745a, miR5260, miR2111m-3p, miR169l-5p, miR398b, and miR398c, participated in negative regulation between miRNAs and circRNAs also involved in the negative regulation of lncRNAs. It indicated that circRNA might acting as multi miRNA sponger competing with different lncRNAs.

### 2.6. Construction of ceRNA-miRNA-Target Genes Regulatory Networks

**Figure 5:**
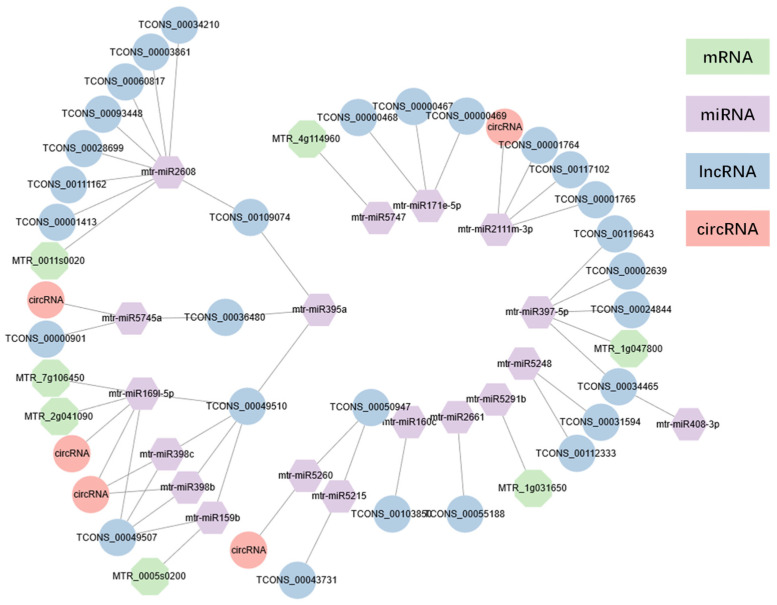
mRNA–miRNA–ncRNA expression network. Lines were based on the negative regulation of miRNA with its target genes.

Based on the negative relationship between miRNA and target genes, a ceRNA regulation network was established (Figure 5). At D0, mtr_miR397-5p negatively regulated four DElncRNAs (*TCONS_00024844, TCONS_00002639, TCONS_00034465, TCONS_00119643*). Within D1 treatment, mtr-miR2608 expression was negatively contrary with *MTR_0011s0020* and eight lncRNAs (*TCONS_00028699, TCONS_00111162, TCONS_00093448, TCONS_00034210, TCONS_00109074, TCONS_00001413, TCONS_00003861, TCONS_00060817*). Under D2 treatment, mtr_miR159b, and mtr_miR169l-5p, constructed mRNA–miRNA–lncRNA(–circRNA) networks, respectively. Intriguingly, mtr_miR159b, mtr_miR398b, mtr_miR398c, and mtr_miR169l-5p had a negative regulatory relationship with *TCONS_00049510* and *TCONS_00049507*. These two lncRNAs belonged to splice variants of DElncRNA genes. Meanwhile, these indicated that both *TCONS_00049510* and *TCONS_00049507* comprised disparate MREs, which interacted with different miRNAs. These could account for the similar expression pattern of mtr_miR169l-5p, mtr_miR398b, and mtr_miR398c. Besides mRNA *MTR_7g106450* and *MTR_2g041090*, and mtr_miR169l-5p were negatively regulated by 2 circRNAs, *mtr_circ_0000090*, and *mtr_circ_0000202*. Strikingly, *MTR_7g106450* (*MtNF-YA2*) and *MTR_2g041090* (*MtNF-YF3*) were presumed to be nuclear transcription factor Y subunit A (NF-YA) family members, which were consistent with previous study acting as target genes of miR169 [29,30,31,32,33]. Mtr_miR169l-5p together with its target mRNA and lncRNA were validated by qRT-PCR, confirming their negative correlated expression pattern (Figure S7). The mtr_miR169l-5p ceRNA network expression in qRT-PCR verified our results in RNA-seq analysis.

Different *mtr_circ_0000090*, and *mtr_circ_0000202* MREs indicated that both of these circRNAs had diverse miRNA MRE sites of miRNA mtr_miR398b and mtr_miR398c. Mtr_miR159b took part in negative regulation of *MTR_0005s0200*, whereas mtr_miR397-5p was participant in negative regulation of *MTR_1g047800* in D2. In consideration of the interaction between mtr_miR397-5p and its negative regulation lncRNAs under D0 condition, mtr_miR397-5p was possibly suppressed by lncRNA in D0 treatment and inhibited expression of *MTR_1g047800* in the drought stress.

## 3. Discussion

### 3.1. SNF-Triggered Pathways Enhanced Drought Tolerance in M. truncatula

Our results showed that AN and IN plants had improved drought tolerance than NN. Especially, AN plants exhibited the optimal drought-resistant properties via SNF-triggered pathway. Based on the RNA-seq analysis, we clarified the potential molecular mechanism of drought tolerance in *M. truncatula* of different nodule treatments.

With D0 treatment, the DEGs in IN were enriched in proline biosynthetic and positive regulation of transcription. Plants accumulated proline in response to abiotic stress of drought [34], heat [35], cold [36], and salt [37]. Hydroxyproline produced by hydroxylation of proline existed in a variety of plant proteins, especially related to cell wall formation and modifications [38]. Cell wall structure modifications enhanced the drought tolerance to osmotic stress [39]. Thus, the proline biosynthesis could promote drought tolerance of IN plants in contrast to NN. Differently from IN plants, the AN DEGs were enriched in response to abiotic stress. The initiation of symbiotic nitrogen fixation was correlated with the legume defense system [40]. Thus, we considered that SNF-triggered response to stress in D0 could improve tolerance to water deficiency.

More defense-responsive genes were expressed to improve the drought tolerance of AN plants in D2 conditions. Severe drought stress-triggered different dynamic regulatory networks in *M. truncatula*. The AN plants specifically showed negative regulation in valine, leucine, and isoleucine degradation; nitrogen metabolism metabolic pathways; and positive adjustment in N-glycan biosynthesis and systemic acquired resistance. In exchange for the reduced nitrogen from the bacteria, the host plant provided the rhizobium with carbon as energy in exchange for the nitrogen from the nodulation [41]. Due to the negative effects on nitrogen metabolism during drought stress [42], carbon sources consumption of *M. truncatula* in SNF was decreased to reserve energies in response to water deprivation. Both abiotic stress and biotic stress with pathogenic or symbiotic bacteria were able to trigger unfolded protein response (UPR) [43,44]. As a fundamental part of glycosylation to ensure protein folding, N-glycans biosynthesis was consequently upregulated in response to UPR. Furthermore, the synthesis of lipid-linked oligosaccharide (LLO) required the sequential addition of sugar residues, which were generated by N-glycans, to the ER lipid dolichol (Dol) [44,45]. Lack of Dol led to lower drought resistance of plants [46]. Altogether, AN plants were more resistant to drought stress through synthesis of N-glycan, systemic acquired resistance due to early effects of SNF-triggered, and reserved energies through reducing metabolism processes.

### 3.2. SNF-Related miRNA Contributed to Drought Tolerance

Regulation of miRNAs was found to be involved in biotic and abiotic stress [47]. In our research, the expression of miRNA contributed to the drought tolerance in AN plants. We found that mtr_miR159b, mtr_miR169l-5p, mtr_miR397, mtr_miR398b, mtr_miR398c, mtr_miR2608, mtr_miR5216, mtr_miR5260, mtr_miR5291, mtr_miR5745a, and mtr_miR5747 were involved in SNF-triggered improvement of drought tolerance. The function of several miRNAs was largely unknown.

SNF might trigger the downregulation of mtr_miR397 and upregulation of its target genes facilitating lignin biosynthesis. Previous studies showed that miR397 was participated in defense response to pathogen infection [48]. Targeted genes of miR397 were involved in lignin biosynthesis and improvement of drought tolerance. Over-accumulated Sv_miR397 in *Arabidopsis* provoked a decrease in lignin content, and was more sensitive to salt stress [49]. Overexpression of *PeLAC10* in *Arabidopsis* led to an increase in the lignin content and improvement of drought tolerance [50]. Moreover, miR397a also affected long-term boron toxicity via its target genes *LAC4* modulating secondary cell-wall biosynthesis in *Citrus sinensis* [51,52]. With *Verticillium dahliae* infection, the ghr-miR397-knockdowned plants exhibited improvement in G-lignin biosynthesis [53]. It indicated that ghr-miR397 target gene *GhLAC4* involved defense-induced lignin biosynthesis. Our early study suggested that rhizobium symbiosis could increase the lignin content in alfalfa [54]. Moreover, miR397 and its target laccase were found to be involved in defense response to *Pythium ultimum* infection [55]. Furthermore, miR397-5p_1 was also reported to mediate the parasitic development of the hemiparasitic plant *Monochasma savatieri* [56]. Laccase acted lignification of root tissues to hamper the pathogen infection and reduce the injury from the pathogen [55]. Therefore, with SNF, miR397-5p displayed lower expression, resulting in the accumulation of target genes that correlated well with the lignification of cell walls, and the incremental lignin content decreased the sensitivity response to drought stress.

The miR398 induced by SNF mediated drought tolerance through the ROS metabolism network. The *CSD*, *APX*, and *CAT*, which were reported as the target genes of sly-miR398b, were involved in SOD, APX, and CAT, respectively [57]. As the target genes of miR398b and miR398c, CSD function was disrupted when infected by the *Bamboo mosaic virus* and accompanying upregulation of miR398 [58]. It suggested that the accumulation of miR398 enhanced tolerance to pathogen infection. Thus, the higher level of mtr_miR398 in IN plants indicated that inactive nodules were more likely to be pathogen infections for plants in the D0 condition. In contrast, the expression of mtr_miR398b and mtr_miR398c in AN plants were lower than NN. It seemed that SNF was able to trigger the downregulation of mtr_miR398. With severe drought stress, mtr_miR398 maintained a lower expression level. Moreover, the overexpression of sly-miR398b enhanced the salinity tolerance in tomatoes [57]. When suffering from water deficit, miR398 was downregulated in response to drought stress [59]. Thus, SNF triggered the miR398 downregulation, resulting in the upregulation of its target genes *CSD*, *APX,* and *CAT* enhancing detoxification of ROS in drought stress.

### 3.3. SNF-Induced ceRNA Network Impact on Drought Tolerance

Our result showed that mtr_miR169l-5p, mtr_miR398b, and mtr_miR398c, had a similar expression pattern. This might be owing to the circRNA and lncRNAs, which had different MREs, absorbing these three miRNAs simultaneously. The miR169 was first demonstrated to target specific NF-YA family members in response to abiotic stress in *Arabidopsis* [60]. It was subsequently determined to be affected in *M. truncatula* root with SNF [29]. With SNF, NY-FA family members were upregulated in a miR169 reduction manner. However, the approach that miR169 content in root affected miR169 accumulation in leave remained largely unknown. Recently study showed that only a few miRNAs were ascribed as high-confidence root-to-shoot mobile candidates in an *Arabidopsis*/*Nicotiana* interfamilial heterograft [61]. We assumed that active nodules of roots could facilitate downregulation of mtr_miR169 in leaves in an unknown manner. As the target genes of miR169, NY-FA family members were accumulated in leaves [31], subsequently activating PEROXIDASE1 expression in response to ROS and improving tolerance to osmotic stress during drought stress [32,62].

**Figure 6:**
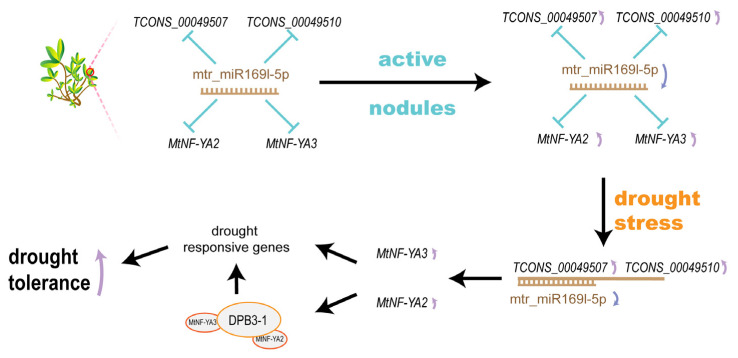
SNF-triggered mRNA–miR169l–lncRNA network in response to drought stress.

In this study, we constructed an mRNA–miR169l–lncRNA dynamic ceRNA network to explain the SNF-triggered plants with improved drought tolerance (Figure 6). We found that mtr_miR169l-5p was in a state of low expression in AN. In the D0 condition, mRNA (*MtNF-YA2* and *MtNF-YA3*) and lncRNA (*TCONS_00049507* and *TCONS_00049510*), as the target genes, were suppressed because of the high expression level of mtr_miR169l-5p. During D1 treatment, sharply increased expression levels of *TCONS_00049507* and *TCONS_00049510* competitively combined with mtr_miR169l-5p led to the upregulation of *MtNF-YA2* in plants. The mtr_miR169l-5p was competitively combined with the lncRNAs in IN and AN plants, while its target genes (*MtNF-YA2*, *MtNF-YA3*) were upregulated. Even though mtr_miR169l-5p was competitively combined by the increasing lncRNA, *MtNF-YA3* was still downregulated by the suppression of the active mtr_miR169l-5p in NN plants. Under D2, due to the competitive interaction between *TCONS_00049507*/*TCONS_00049510* and mtr_miR169l-5p, plants were able to continuously upregulated *MtNF-YA2* and *MtNF-YA3* in AN plants.

It was reported that NF–YA families participated in drought response by mediating the expression of several drought stress-responsive genes in an ABA-dependent manner [63,64]. Overexpression of *GmNF-YA5*, *NF-YA8*, and *GmNFYA13*, enhanced the drought tolerance of plants [62,65,66]. Meanwhile, *NF-YA2*, *NF-YB3*, and DPB3-1 could form a transcriptional complex to activate the promoter of the heat stress-inducible gene in *Arabidopsis* [67]. Therefore, *MtNF-YA2* and *MtNF-YA3* were accumulated through SNF-induced downregulation of mtr_miR169l-5p, and the combination between mtr_miR169l-5p and *TCONS_00049507*, which were rapidly upregulated.

## 4. Materials and Methods

### 4.1. Plant Materials and Treatments

*M. truncatula* seeds were disinfected with 75% alcohol for 10 min and then vernalized at 4 °C for 48 h in petri dishes with wet filter paper in the darkness. We irrigated the sands with diluted NaClO (1000 mg/L) before planting seedlings to kill the potential rhizobia in sand or on the pots. And the results showed that seedlings inoculated with rhizobia developed active nodules, whereas the uninoculated seedlings did not develop any nodules. After germinating in the plant growth chamber for 3 days, the 1.5–2.5 cm seedlings were transplanted into 9 cm plots with sterilized sand (100 mesh) in greenhouse of Northwest A&F University, Yangling, Shaanxi, China (108.07° E, 34.29° N). The reformative 1/2 Hoagland solution [68] was irrigated every two days with 16 h illumination in 24 °C and 8 h darkness in 20 °C.

The 4 cm-aboveground seedlings were stochastically divided into 3 groups (1) no nodules (uninoculated, NN) group without rhizobia inoculated, (2) inactive nodules (IN) group with ‘Duomeng’ rhizobia inoculant (CLOVER, Beijing, China) inoculated but irrigated with full N 1/2 Hoagland solution to inactivated nodules, and (3) activate nodules (AN) group with rhizobia and irrigated with low N (0.25 mM NO_3_^−^) 1/2 Hoagland solution. After 60 days since inoculation, each group (NN, IN, AN) was subjected to different degrees of drought stress. Sand was carefully removed from the roots. Seedlings were transferred to pots, which were filled with dry sterilized sand for drought treatments. Plants before drought treatment were set as control (D0). Seedlings planted in dry sand for 3 and 8 h were defined as mild (D1) and severe (D2) drought treatments, respectively. Thus, nine treatments were obtained, D0_NN, D0_IN, D0_AN, D1_NN, D1_IN, D1_AN, D2_NN, D2_IN, D2_AN (Table 1). Leaves from 3 seedlings randomly selected were mixed as one repetition and 3 repetitions were performed per treatment, namely nine pots of seedlings for each treatment. Samples were stored in liquid nitrogen immediately for RNA extraction and subsequent analysis.

### 4.2. Transcriptome Sequencing

The RNA was extracted with Hipure Total RNA Mini Kit (Magen, Shanghai, China). 10 μg extracted RNA was removed rRNA and generated mRNA, lncRNA, and circRNA libraries with Ribo-off rRNA Depletion Kit (Vazyme, Nanjing, China). The small miRNA libraries were generated by Multiplex Small RNA Library Prep Kit for Illumina (NEBNext, Ipswich, MA, America). The prepared libraries were sequenced on novaseq of Illumina.

### 4.3. Different Expression Pattern of Transcriptome

The raw sequencing data were evaluated by FAST-QC (http://www.bioinformatics.babraham.ac.uk/projects/fastqc/, accessed on 27 July 2021). HISAT2 [69] was employed in mapping the RNA-seq data to the *M. truntacula* genome (https://www.ncbi.nlm.nih.gov/genome/?term=MedtrA17_4.0, accessed on 5 May 2021). Bowtie2 [70] was used to map the RNA-seq data to obtain novel lncRNAs. BWA algorithm [71] was used to map the filtered clean reads to the miRbase and Rfam database, and subsequently prediction of novel miRNA. ACFS2 [72] was employed in prediction of novel circRNAs.

Bam documents obtained from mapping to reference genome were transcript reconstructed by StringTie [73]. After filtering the coding ability of the reconstructed transcript was predicted by CPAT [74] and the length of the sequence, the novel lncRNA information would be acquired. FPKM and TPM which can eliminate the influence of gene length and sequencing amount for calculating gene expression were used to compare differential expressed genes among different samples. DESeq [75] algorithm was selected to screening differentially expressed mRNA and lncRNA, with threshold logFC > 1 or logFC < −1, *p*_value < 0.05, false discovery rate (FDR) < 0.05 [76]. The analysis was divided into miRNAs mapping to the *M. truncatula* genome and novel miRNAs that were mapped to module species or predicted by biological software. EBSeq [77] algorithm was selected to screen differentially expressed miRNAs with threshold logFC > 0.585 or logFC < −0.585, FDR < 0.05. The predicted circRNAs expressed in different samples with diverse rpkm were selected as differentially expressed circRNAs.

### 4.4. Feature Analysis of Transcriptome

Gene Ontology (GO) enrichment analysis was applied to analyze the main function of the differential expression genes according to the gene ontology, which is the key functional classification of NCBI [78]. Generally, Fisher’s exact test and *χ*^2^ test were used to classify the GO category, and the FDR was calculated to correct the *p*-value. The smaller the FDR, the small the error in judging the *p*-value.

Pathway analysis was used to find out the significant pathway of the differential genes. Pathway annotations of Microarray genes were downloaded from KEGG (http://www.genome.jp/kegg/, accessed on 30 July 2021). The fisher exact test was used to find the significant enrichment pathway [79,80]. The resulting *p* values were adjusted using the BH FDR algorithm with FDR < 0.05.

Following different signal density change tendencies of genes under different situations, we identified a set of unique model expression tendencies. TCseq (https://bioconductor.org/packages/TCseq/, accessed on 20 August 2021) package was used to preform series cluster analysis of DEGs, DEmiRNAs, DElncRNAs, and DEcircRNAs. WGCNA [81] package was adhibited to identify potential genes associated with traits.

WGCNA package [81] was used to calculate the soft threshold, draw the network heatmap, and module-trait relationship heatmap. Cytoscape [82] with its plug-in cytoHubba [83] was employed to plot hub genes co-expression network.

### 4.5. Identification of ceRNA Network

Negative correlation relative regulations were predicted by plant miRNA target gene prediction algorithm psRNAtarget [27] considering DEGs as research object and miRNA target gene prediction algorithm miranda [84] regarding DElncRNAs and DEcircRNAs as a research object. Cytoscape [82] was used to draw mRNA–miRNA–ncRNA co-expression network.

### 4.6. qRT-PCR Verification

Total RNA was extracted by EasyPure miRNA Kit (TransGen, ER601-01), and reverse transcribed by *Evo M-MLV* RT Kit with gDNA Clean for qPCR II (Accurate Biotechnology (Hunan) Co., Ltd., AG11711, Changsha, Hunan, China). Reverse transcriptional cDNA was subsequently mixed with SYBR^®^ Green Premix Pro Taq HS qPCR Kit (Accurate Biotechnology (Hunan) Co., Ltd., AG11701). The primers used were listed in Table S13. Expression levels of the *M. truncatula* actin gene and U6 gene were used to normalize the expression levels of select mRNA, lncRNAs, and miRNA, respectively. The relative expression was then analyzed via the 2^−ΔΔCT^ method [85]. Data analysis was charted in Excel.

## 5. Conclusions

Taken together, SNF-triggered plants response to stress might trigger the improvement of tolerance to water deficiency. *M. truncatula*-forming nodules exhibited improved drought tolerance compared with the NN group. The decrease of mtr_miR169l-5p, mtr_miR398b, and mtr_miR398c, provided an opportunity for the improvement expression levels of lncRNAs and mRNAs. As the water deficiency became severe, plants reserved energy and medicated systemic acquired resistance due to early effects of SNF-triggered to improve the drought tolerance. We constructed a ceRNA competing network that the downstream drought-response genes were upregulated continuously in AN plants, while *TCONS_00049507* and *TCONS_00049510* competitively interacted with mtr_miR169l-5p. Liberated *MtNF-YA2* and *MtNF-YA3* subsequently participated in the downstream response to drought. Our results explained that the SNF impacted drought tolerance with molecular pathways, and the mRNA–miR169l–ncRNA ceRNA network was constructed to promote the genetic research and provide agronomic character improvement theoretical grounding in legumes.

## Figures and Tables

**Table 1:** Nine grouping information with different drought and nodule treatments.

Treatment	Without Drought (D0)	Mild Drought (D1)	Severe Drought (D2)
**No Nodule (NN)**	D0_NN	D1_NN	D2_NN
**Inactive Nodule (IN)**	D0_IN	D1_IN	D2_IN
**Active Nodule (AN)**	D0_AN	D1_AN	D2_AN

**Table 2:** miRNA negatively regulated target mRNA, lncRNA, circRNA.

Treatment	miRNA	mRNA	lncRNA	CircRNA
**D0**	mtr-miR2661		*TCONS_00055188*	
	mtr-miR397-5p		*TCONS_00119643*	
			*TCONS_00034465*	
			*TCONS_00002639*	
			*TCONS_00024844*	
	mtr-miR408-3p		*TCONS_00034465*	
	mtr-miR5215		*TCONS_00043731*	
	mtr-miR5248		*TCONS_00031594*	
			*TCONS_00112333*	
D1	mtr-miR2608	*MTR_0011s0020*	*TCONS_00060817*	
			*TCONS_00003861*	
			*TCONS_00001413*	
			*TCONS_00109074*	
			*TCONS_00034210*	
			*TCONS_00093448*	
			*TCONS_00111162*	
			*TCONS_00028699*	
D2	mtr-miR159b	*MTR_0005s0200*	*TCONS_00049507*	
			*TCONS_00049510*	
	mtr-miR169l-5p	*MTR_7g106450*	*TCONS_00049507*	mtr_circ_0000090
		*MTR_2g041090*	*TCONS_00049510*	mtr_circ_0000202
	mtr-miR397-5p	*MTR_1g047800*		
	mtr-miR5291b	*MTR_1g031650*		
	mtr-miR5747	*MTR_4g114960*		
	mtr-miR160c		*TCONS_00103850*	
	mtr-miR171e-5p		*TCONS_00000469*	
			*TCONS_00000468*	
			*TCONS_00000467*	
	mtr-miR2111m-3p		*TCONS_00001765*	mtr_circ_0000167
			*TCONS_00001764*	
			*TCONS_00117102*	
	mtr-miR395a		*TCONS_00036480*	
			*TCONS_00109074*	
			*TCONS_00049510*	
	mtr-miR398b		*TCONS_00049507*	mtr_circ_0000202
	mtr-miR398c		*TCONS_00049510*	
	mtr-miR5215		*TCONS_00050947*	
	mtr-miR5260		*TCONS_00050947*	mtr_circ_0000037
	mtr-miR5745a		*TCONS_00000901*	mtr_circ_0000112
			*TCONS_00036480*	

## Data Availability

The data have been submitted to the China National GeneBank DataBase (CNGBdb, https://db.cngb.org/) with project number CNP0002711.

## Supplementary Materials

The following supporting information can be downloaded at: https://www.mdpi.com/article/10.3390/ijms232214237/s1.

## Abbreviations

NN	no nodule treatment
IN	inactive nodule treatment
AN	active nodule treatment
D0	no drought stress treatment
D1	mild drought stress treatment
D2	severe drought stress treatment
ceRNAs	competing endogenous RNAs
SNF	symbiotic nitrogen fixation
circRNAs	circular RNAs
lncRNAs	long noncoding RNAs
sRNAs	small noncoding RNAs
miRNAs	microRNAs
siRNAs	small-interfering RNAs
DEGs	differentially expressed genes
DElncRNAs	differentially expressed lncRNAs
DEcircRNAs	differentially expressed circRNAs
DEmiRNAs	differentially expressed miRNAs
pri-miRNA	primary-miRNA
Pol II	RNA polymerase II
pre-miRNA	precursor-miRNA
DCL1	Dicer-like protein 1
RISC	RNA-induced silencing complex
AGO	Argonaute
MREs	miRNA response elements
NF-Y	NUCLEAR FACTOR-Y
NF-YA	nuclear transcription factor Y subunit A
GO	gene ontology
KEGG	Kyoto Encyclopedia of Genes and Genomes
WGCNA	weighted gene co-expression network analysis
LLO	lipid-linked oligosaccharide
Dol	dolichol
FDR	false discovery rate
