# Supplementary material for: Identification of Competing Endogenous RNAs (ceRNAs) Network Associated with Drought Tolerance in Medicago truncatula with Rhizobium Symbiosis
Source: Int J Mol Sci. 2022 Nov 17;23(22):14237. doi: 10.3390/ijms232214237 (PMC9696283; doi:10.3390/ijms232214237)
Supplement: Supplementary file 1 [file ijms-23-14237-s001.zip › Supplementary_figure.pdf]

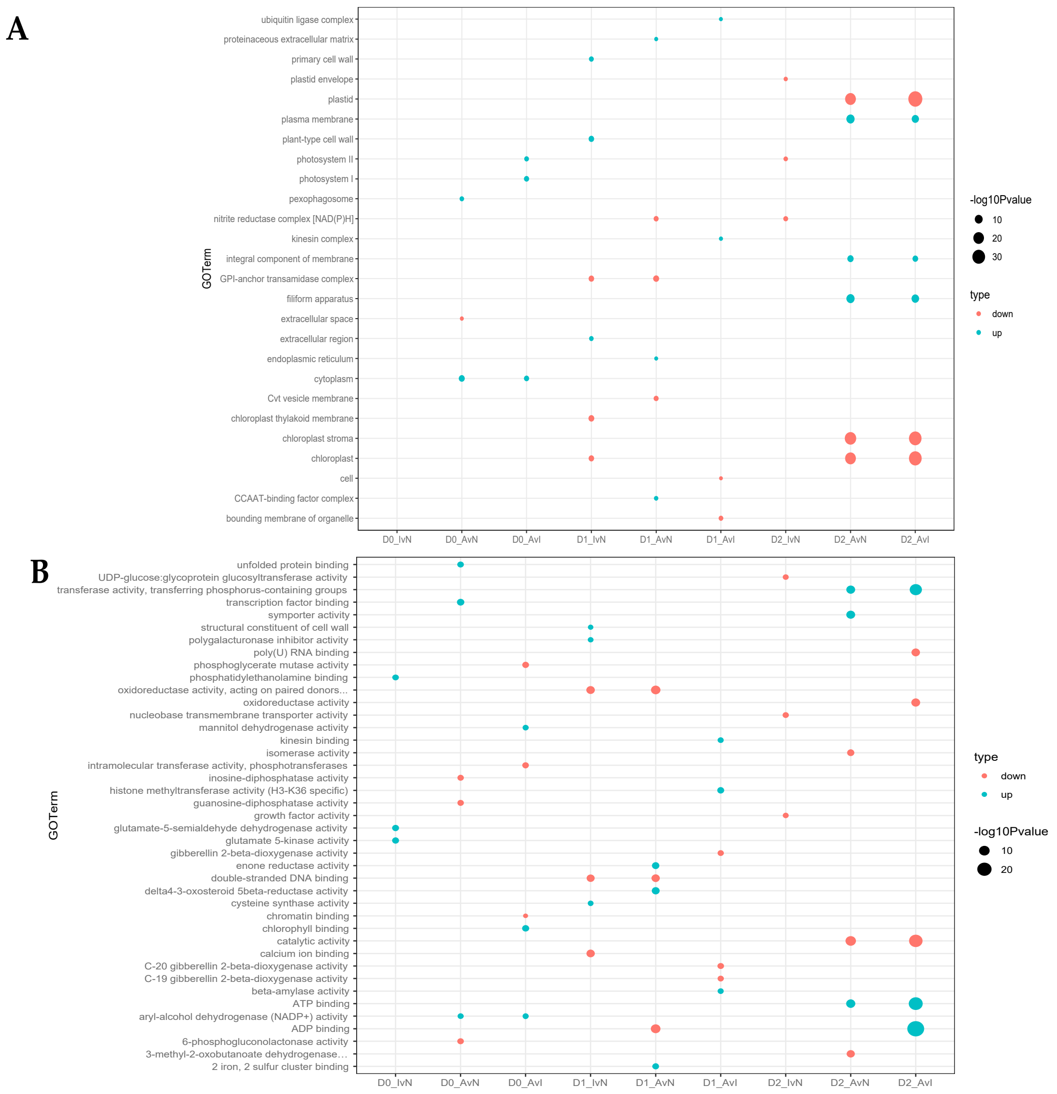

Figure S1. The mRNA GO analysis under SNF and drought stress. GO analysis of (A) cellular component (CC) and (B) biological process (BP) under SNF and drought stress. All the GO terms and pathways were filtrated with P-value < 0.05. Top 3 GO terms/pathways in up-regulated or down-regulated regulation module for each sample with the lowest P-value were selected.

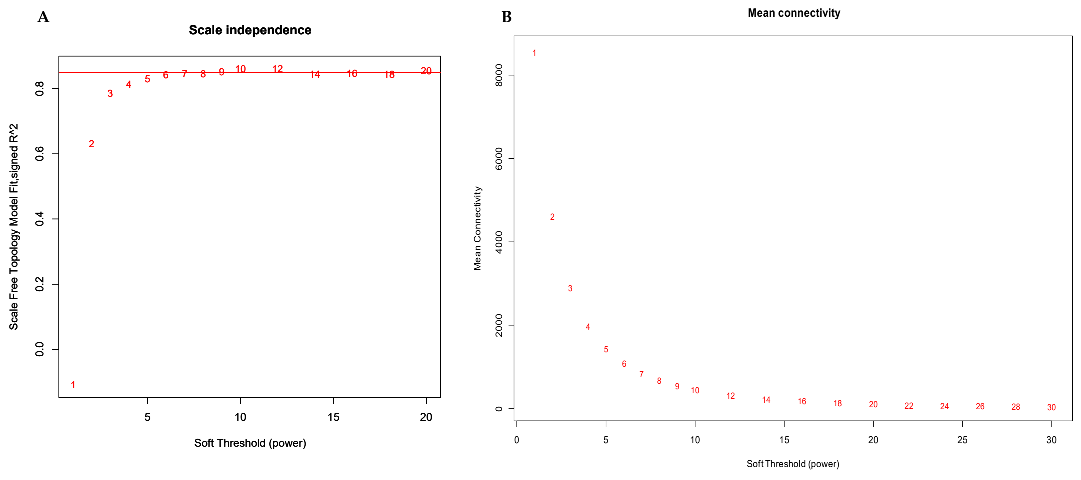

Figure S2. Soft threshold calculation in WGCNA analysis. (A) Evaluation parameters of scale-free networks; (B) Soft threshold and average connectivity.

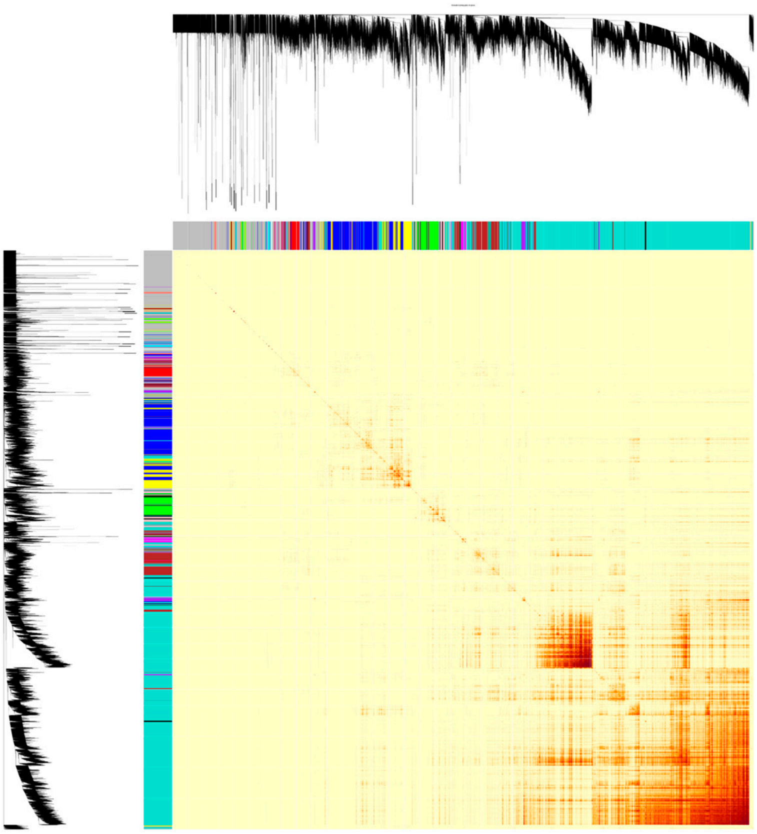

Figure S3. The DEGs of different modules hierarchical cluster network heatmap.

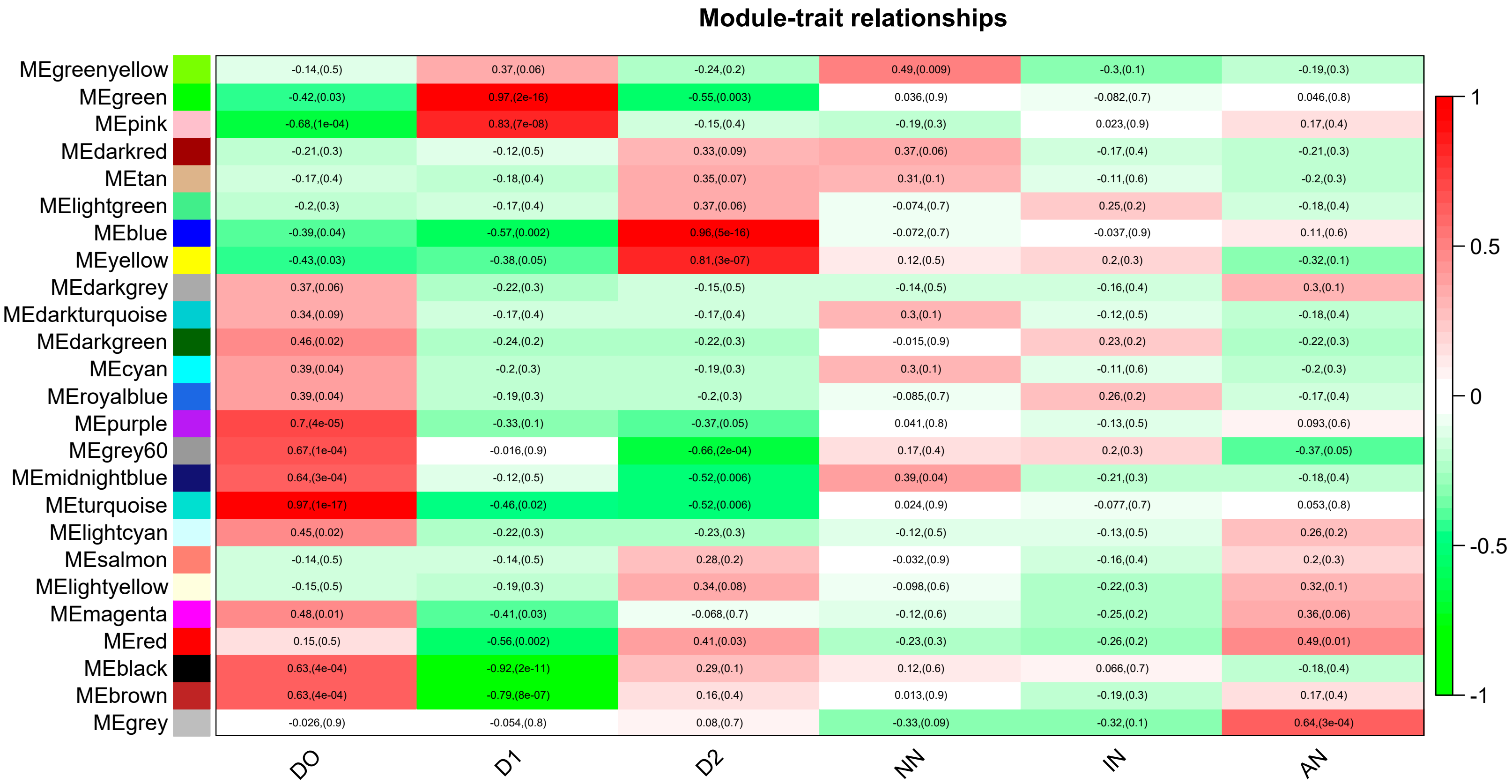

Figure S4. Module-trait relationships of WGCNA analysis. DEGs in different modules were evaluated the correlation with the treatments of without drought (D0), mild drought (D1), severe drought (D2), no nodules (NN), inactive nodules (IN), active nodules (AN).

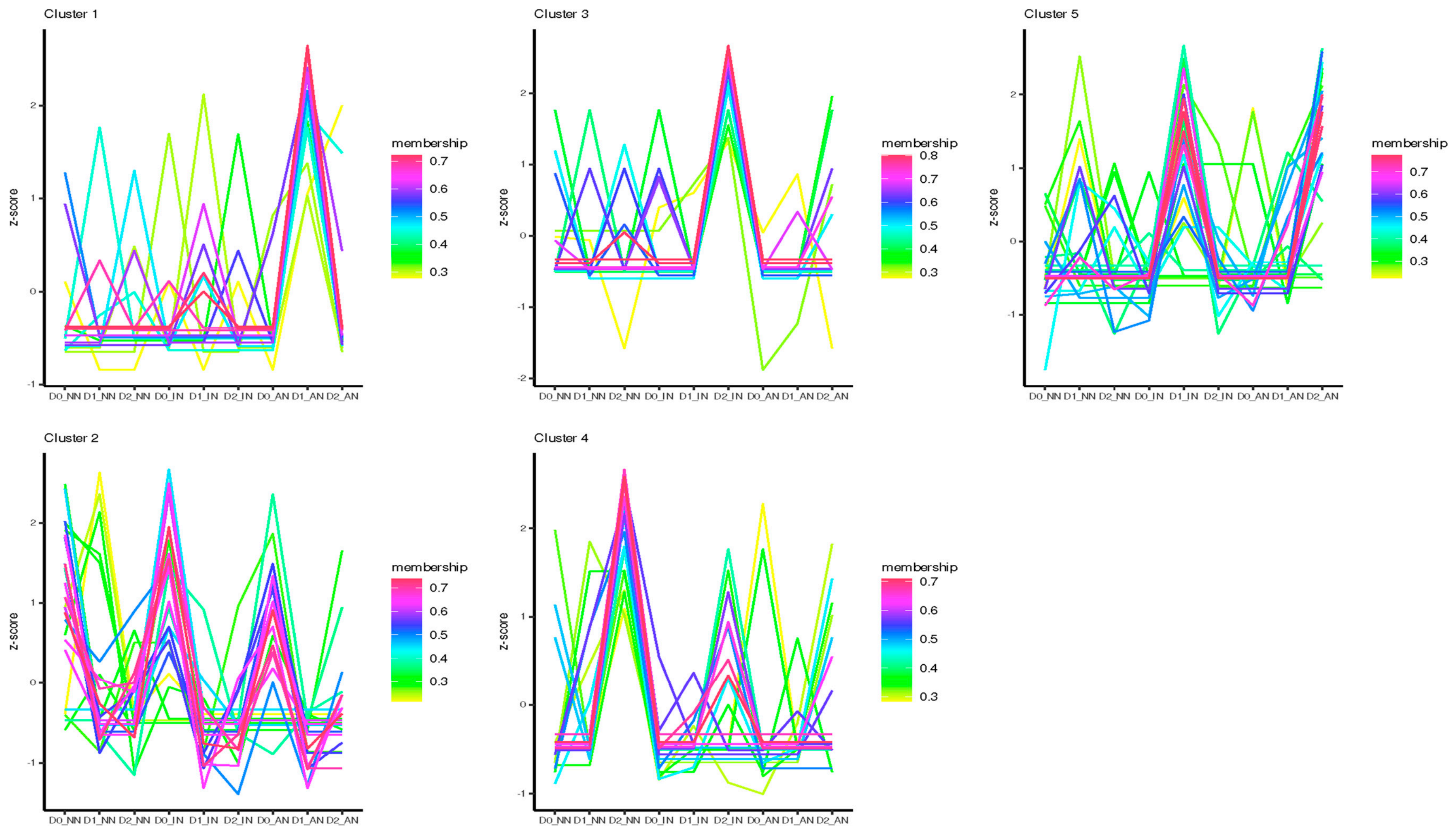

Figure S5. The circRNA cluster prediction analysis.

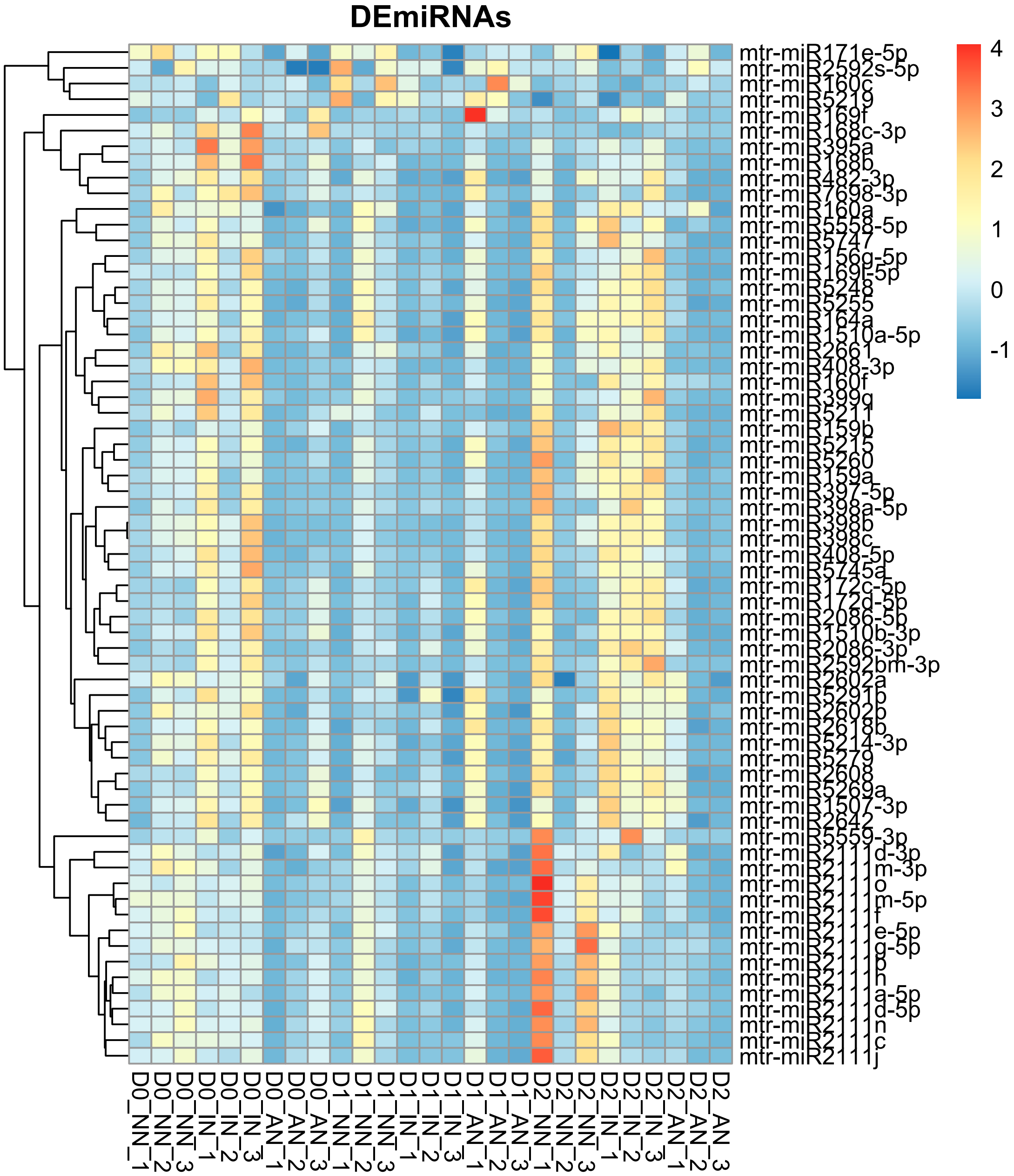

Figure S6. The DEmiRNA expression cluster heatmap.

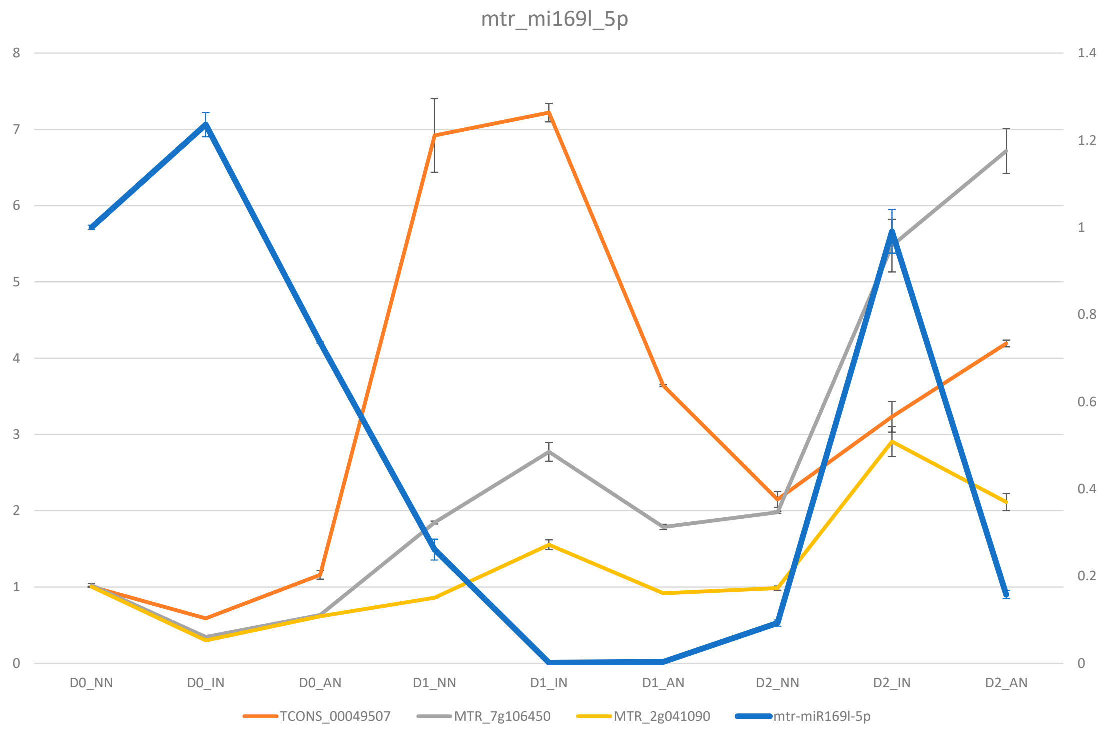

Figure S7. The qRT-PCR of mtr_miR169l-5p ceRNA network. To visual exhibit the expression trends of ceRNA network, the mtr_miR169l-5p expression was plotted according to the right coordinate system.
